# Overlapping and distinct intrinsic connectivity of hippocampal and triple networks in autistic individuals with and without a comorbid condition of ADHD

**DOI:** 10.3389/fpsyt.2026.1894578

**Published:** 2026-09-03

**Authors:** Lang Chen, Anna Riggs, Nickolas Luckenbach

**Affiliations:** 1 Neuroscience program, Santa Clara University, Santa Clara, CA, United States; 2 Department of Psychology, Santa Clara University, Santa Clara, CA, United States

**Keywords:** ADHD, ASD, comorbidity, functional connectivity, hippocampus

## Abstract

Autism Spectrum Disorder (ASD) and Attention-Deficit/Hyperactivity Disorder (ADHD) are conventionally thought to be very distinct neurodevelopmental disorders, but many autistic individuals also exhibit symptoms of ADHD. Extensive research has focused on examining the behavioral profiles of autistic individuals with comorbid ADHD, but their neurobiological profiles remain understudied. The current study aimed to examine the intrinsic connectivity of the hippocampal memory, default-mode, central executive, and salience networks in autistic individuals with or without ADHD. Using data from the Autism Brain Imaging Data Exchange (ABIDE I), we identified 23 individuals with autistic disorder and comorbid ADHD (Comorbid), 23 individuals with autistic disorder only (ASD-only), and 23 non-autistic individuals (non-ASD), who were well-matched on gender, age, and full-scale IQ. Results showed that the Comorbid group showed hypo-connectivity of the hippocampal memory, DMN, CEN, and SN nodes compared to the ASD-only group. In addition, the connectivity of the right hippocampus and PCC showed distinct relationships with the ASD symptom severity in autistic individuals with and without ADHD. These findings indicated that the comorbid condition has a distinct neurobiological profile compared to the ASD-only group, suggesting that future intervention and education should have specialized programs to support affected individuals with a focus on their memory functions in addition to their socio-communicative challenges.

## Introduction

Autism Spectrum Disorder (ASD) and Attention-Deficit/Hyperactivity Disorder (ADHD) are both neurodevelopmental disorders that are conventionally thought to be distinct, with ASD characterized by deficits in socio-communicational skills as well as repeated, restricted interest and behavior (1, 2), while ADHD characterized by inattention, trouble focusing, hyperactivity and impulsivity (1). Since the dual diagnosis was available in DSM-5, researchers have found high comorbidity of ASD and ADHD (3–6), and some even suggested that 50%~70% of individuals diagnosed with ASD also exhibit symptoms of ADHD (7). The comorbid condition has posed significant challenges to the current clinical and educational practice (6, 8, 9). Since the current literature suggests that both ASD and ADHD are characterized by aberrant brain connectivity (10–13), our study aims to examine the resting-state functional connectivity (RSFC) in ASD with or without a comorbid condition of ADHD, and adjudicate between different theoretical models of the comorbid condition of ASD and ADHD.

Mounting evidence from existing functional neuroimaging studies (14–17) has suggested that ASD and ADHD may be associated with atypical functional activations and connectivity, especially in three well-known brain networks (i.e., the triple networks), namely, the default mode network (DMN), central executive network (CEN), and the salience network (SN; 10, 11, 18–20). Typically, the DMN consists of multiple brain regions, including the ventromedial prefrontal cortex (vmPFC) and posterior cingulate cortex (PCC), for their roles in internal mental processes such as daydreaming, introspection, mentalizing, self-referential processing, and social evaluation (18, 21). The CEN conventionally refers to prefrontal and parietal regions for their distinct roles in decision making, goal-directed behavior, problem solving, and working memory (22, 23). The SN, mostly anchored in the anterior insula, is commonly associated with recognition of salient stimuli and switching between other large-scale brain networks such as the DMN and CEN (22, 24). Extensive studies have examined the functional connectivity in ASD, and revealed under-connectivity in ASD compared to non-ASD of DMN nodes (14, 17, 25), aberrant functional connectivity of CEN in ASD (26), overconnectivity between CEN and DMN (15), as well as aberrant connectivity of SN nodes in ASD (20, 27, 28). Similarly, extensive studies have also linked the aberrant functional connectivity in triple-networks in ADHD (29–35). Thus, our study will focus on the functional connectivity of the nodes in the triple networks in ASD with and without comorbid ADHD.

**Figure 1:**
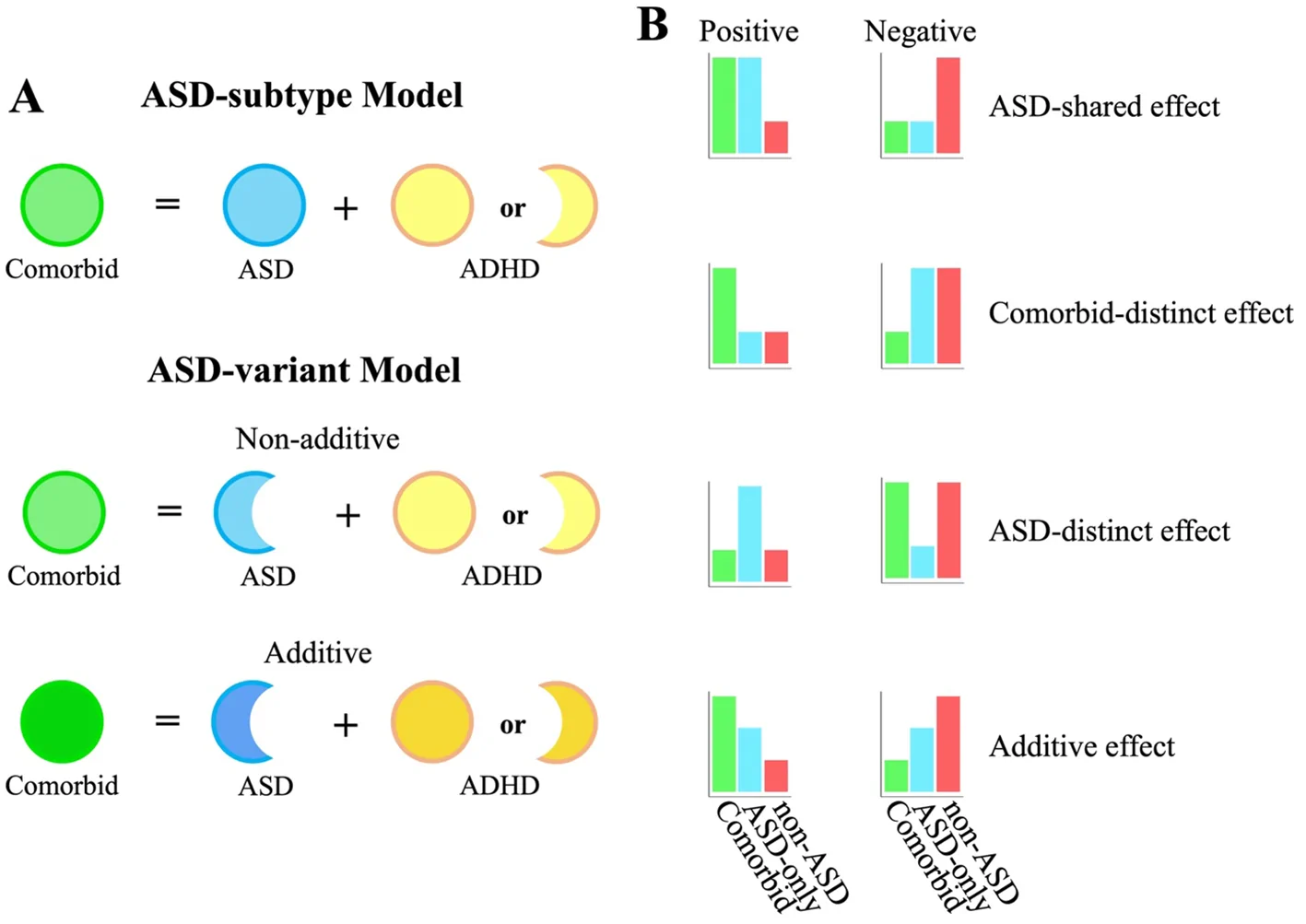
Theoretical models of ASD with a comorbid ADHD condition and their corresponding predictions. **(A)** The ASD-subtype vs. two ASD-variant (non-additive and additive) models of ASD with a comorbid ADHD condition, and **(B)** the illustrations of model predictions for potential observed effects comparing the Comorbid (ASD+ADHD), ASD-only, and non-ASD groups. The ASD-subtype model suggests that autistic individuals with comorbid ADHD are one subtype of ASD, so that the dysfunctions are comparable in the Comorbid and ASD-only groups (ASD-shared effect), but we should also observe the Comorbid-distinct effect due to their additional ADHD symptoms. The ASD-variant models, on the contrary, propose that the Comorbid condition does not completely overlap with the ASD condition. Therefore, the ASD-only group shows its distinct effect (ASD-distinct effect) compared to the Comorbid group. Depending on whether the additional ADHD condition increases symptom severity in affected individuals or not (Additive effect), the ASD-variant model can be non-additive or additive. The positive and negative patterns of hypothetical effects are determined by comparing the Comorbid and ASD-only groups to the non-ASD group.

The existing literature on ASD with comorbid ADHD remains impoverished, and the brain correlates are yet to be characterized (36). Studies have shown that the comorbid group has shared behavioral challenges and neurobiological underpinnings as the ASD-only group (7, 36), but also distinct behavioral, structural, or functional patterns (37–44). The inconsistent results lead to competing models to understand the difference between autistic individuals with or without comorbid ADHD. The first model is the ASD-subtype model (**Figure 1A**), suggesting that the Comorbid group (autistic individuals with ADHD) is simply a subtype of ASD: they fully share neurobiological correlates with the ASD-only group (i.e., ASD-shared effect in **Figure 1B**) in addition to ADHD-relevant neurobiological effects compared to the ASD-only group (i.e., Comorbid-distinct effect in **Figure 1B**). Some studies have indeed suggested that brain activations in social tasks were found to be comparable for the Comorbid and ASD-only groups (40), and there were no group differences for the resting-state functional connectivity network structure at the global level (45) and within the DMN (46). Thus, it is possible that the Comorbid group can be considered as a subtype of ASD with additional clinical conditions. By contrast, some existing findings suggest an alternative model, namely, the ASD-variant model (**Figure 1A**), and argue that the Comorbid and ASD-only groups were not fully comparable. For example, Tye et al. (47) showed shared atypical ERP signatures in a Go/No-Go task for the Comorbid and ASD-only groups. Still, the ASD-only group showed distinct ERP features compared to the Comorbid and non-ASD participants (i.e., an ASD-distinct effect in **Figure 1B**). A similar ASD-distinct effect was observed in the primary somatosensory and motor circuit anatomy as well (48). Similarly, a recent study also revealed distinct RSFC patterns in the Comorbid and ASD-only groups compared to the non-ASD group, and the Comorbid group showed overall decreased functional connectivity compared to the ASD-only group (46). These findings suggest that the Comorbid and ASD-only groups may share a neurobiological basis (i.e., the ASD-shared effect in **Figure 1B**), but they are also characterized by distinct features on their own (i.e., the ASD-distinct and Comorbid-distinct effects in **Figure 1B**). Thus, autistic individuals with and without comorbid ADHD showed qualitatively distinct patterns of underlying neurobiological underpinnings.

In a different vein, it remains unclear whether the comorbid condition intensifies the clinical or neurobiological dysfunctions in affected individuals (Non-additive vs. Additive ASD-variant models in **Figure 1A**). Behavioral studies have predominantly revealed worse ASD symptoms and cognitive functions in the Comorbid group compared to the ASD-only group (4, 49–51), but studies also reported no group differences in social functions (5, 52). The neuroimaging literature was also diverging. Some evidence has shown that the Comorbid and ASD-only groups had differential activations during cognitive tasks (37, 42, 48), supporting an additive ASD-variant model (**Figure 1A**). Other studies, however, found no group difference in brain activation during a social task (40), resting-state neurophysiological activity (53), or brain structures (48), implying a non-additive ASD-variant model (**Figure 1A**).

In the current study, we examined the difference in RSFC in the triple networks between the Comorbid and ASD-only groups to address these literature gaps. In addition, we extended the literature by including the hippocampal memory network in this study because recent studies have suggested that ASD has memory challenges (54–56) and abnormal connectivity of the hippocampal memory system (57–60). Specifically, we compared the connectivity of key brain regions in DMN, CEN, SN, and bilateral hippocampal nodes of autistic individuals with comorbid ADHD (Comorbid group) to autistic individuals without comorbid ADHD (ASD-only), as well as a group of individuals without either clinical condition (non-ASD). We examined the presence of various effects (**Figure 1B**) to test the proposed theoretical models, namely, ASD-subtype vs. ASD-variant models. In addition, we also explored the association between the RSFC of these network nodes and ASD symptoms. With a theory-driven approach, we could clarify the important neurobiological models of the comorbid condition of ASD and ADHD in brain networks that are crucial to understanding these neurodevelopmental conditions (10, 11, 18).

## Methods

### Participants

All the participants were selected from an open-access dataset, the Autism Brain Imaging Data Exchange (ABIDE I), from two sites (NYU and KKI), since these two sites provide specific information about comorbid conditions. In total, 23 participants with a comorbid ASD+ADHD condition were chosen, and an additional 23 ASD-only and 23 non-ASD individuals were selected with an in-house script to match the three groups (Comorbid, ASD-only, and non-ASD) on gender, age, full-scale IQ, distribution across two sites, and their movement parameters (i.e., framewise displacement; FD) in the resting-state fMRI scan. To ensure the quality of the fMRI data, the framewise displacement of all scans was also kept below 0.5 mm. The detailed demographic information and their movement parameters can be seen in **Table 1**.

**Table 1:** The demographic, cognitive and symptomatic information of all participants.

Variable names	Comorbid (n = 23)		ASD-only (n = 23)		Non-ASD (n = 23)		*χ* ^2^	*p*
Gender (F:M)	5	18	4	19	5	18	0.179	0.914
Site (NYU: KKI)	15	8	17	6	17	6	0.563	0.755
	Mean	SD	Mean	SD	Mean	SD	*F*	*p*
Age	11.99	6.99	13.25	5.05	13.14	4.56	0.355	0.702
Full-scale IQ	108.48	21.15	109.48	15.67	114.96	12.82	0.979	0.381
Mean FD (mm)	0.13	0.09	0.12	0.10	0.10	0.07	0.669	0.516
ADI-R*								
Social	18.86	5.78	19.19	5.29			0.038	0.846
Verbal communication	15.10	5.09	16.10	5.08			0.406	0.527
Restricted, repetitive behavior	5.48	2.80	5.33	2.56			0.030	0.864
ADOS								
Total	10.87	2.91	10.91	3.53			0.002	0.964
Communication	3.61	1.03	3.39	1.37			0.368	0.547
Social	7.26	2.53	7.52	2.41			0.218	0.722
Stereotyped behaviors and restricted interests	2.57	1.16	2.39	1.62			0.176	0.677

*Two participants in the Comorbid and 2 participants in the ASD-only groups had missing data for the ADI-R. NYU, New York University; KKI, Kennedy Krieger Institute; FD, frame-wise displacement; ADI-R, Autism Diagnostic Interview-Revised; ADOS, Autism Diagnostic Observation Schedule.

#### MRI processing and analysis

##### Preprocessing

The resting-state fMRI data from ABIDE I were downloaded and analyzed using SPM12 (61). The first 8 volumes were not analyzed to allow for T1 equilibration. The data were then preprocessed with a standard pipeline, including correction for slice timing, motion realignment, and band filtering (0.008~0.1 Hz). Since the NYU and KKI sites had different scan parameters (e.g., scan sequence and TR), the data from the two sites were preprocessed separately. The individual data were then normalized into MNI152 space and then smoothed with a Gaussian kernel with a FWHM of 6 mm. To keep the minimum of data manipulation, we did not perform white matter and CSF regression or scrubbing besides FD thresholding.

##### ROI selection

Based on the literature (62–64), we chose the following coordinates for DMN, ventromedial prefrontal crotex (vmPFC), -2,38,-12; posterior cingulate cortex (PCC), -6,-44,34; for CEN, bilateral dorsolateral prefrontal cortex (dlPFC) -/+46,20,44 and bilateral posterior parietal cortex (PPC), -40,-56,44 and 52,-52,50; for SN, bilateral fronto-insular cortex (FIC) -34,20,-8 and 39,23,-4, and anterior cingulate cortex(ACC) 6,24,32. In addition, we included the bilateral hippocampus (-/+24,-14,-20) for the hippocampal-memory network.

##### Individual-level and group-level analysis

Using the seed-to-whole brain approach, the mean time series data of each ROI were de-meaned and then regressed on all other voxels in the brain for each participant while the effects of movement parameters on 6 directions (x, y, z, pitch, yaw, and roll) were controlled for. The beta coefficients were then converted to *t* maps for each ROI. At the group level, we compared the t-maps of autistic individuals with a comorbid condition with individuals in the ASD-only and non-ASD groups separately, to reveal the atypical functional connectivity in the Comorbid group. The significantly activated clusters were identified at a height threshold of *p* < 0.01 with family-wise error (FWE) corrections for multiple comparisons at the cluster level of *p* < 0.01 (128 voxels based on Monte Carlo simulations). We also examined whether these clusters could survive a more stringent threshold with a height of *p* <.001 and an FWE-corrected cluster level of *p* < 0.01 (41 voxels based on Monte Carlo simulations, see **Supplementary Table 1**). To provide more comprehensive results, we chose to use the results from the lower threshold in the following analysis.

##### Data analytical approach

Since atypical functional connectivity was revealed separately by comparing the Comorbid and ASD-only groups and Comorbid and non-ASD groups, we classified their patterns according to our hypothesis (**Figure 1B**) on the beta values of all connectivity identified by both comparisons. To further control for any potential difference between the groups, we confirmed the differences between Comorbid, ASD-only, and non-ASD groups in RSFC by using an ANCOVA with gender, age, FSIQ, and FD included as covariates. When the main effect of the diagnosis group was significant (after FDR correction since we ran the ANCOVA on various RSFC beta values simultaneously), *post hoc* tests were conducted to examine pairwise group differences and classify the observed patterns (**Figure 1B**). When significant differences were observed between Comorbid and ASD-only, as well as between ASD-only and non-ASD groups, the additive effect was observed. When the Comorbid and ASD-only groups showed significant differences from the non-ASD group, the ASD shared effect was observed. When the significant difference was found only in the Comorbid group compared to the ASD-only and non-ASD groups, the comorbid distinct effect was observed. When the significant difference was found only in the ASD-only group compared to the Comorbid and non-ASD groups, the ASD-distinct effect was observed. Other significant patterns with a significant main effect of Group were considered as undetermined patterns.

In addition, we further explored whether the observed atypical connectivity could predict ASD symptoms measured by ADI-R and ADOS in the Comorbid and ASD-only groups when the effects of age, gender, FSIQ, and FD were controlled for, and the significance was determined by the FDR-adjusted *p*-values. For connectivity showing ASD-shared effects, we examined the main effect of connectivity beta values predicting ADI-R subscale scores, but for connectivity showing ASD-distinct or Comorbid-distinct effects, we focused on an interaction between Group and connectivity beta values on ASD symptom scores measured by ADI-R.

## Results

### Behavioral analysis

With careful matching, the ASD+ADHD group did not differ in age, gender, and full-scale IQ from the ASD-only and TD groups (all *p*s > 0.381; **Table 1**). Furthermore, after the matching, the ASD+ADHD did not differ from the ASD-only group on the ASD symptoms measured by the ADOS (2) and ADI-R (65).

#### Atypical connectivity in the comorbid group compared to the ASD-only and non-ASD groups

**Figure 2:**
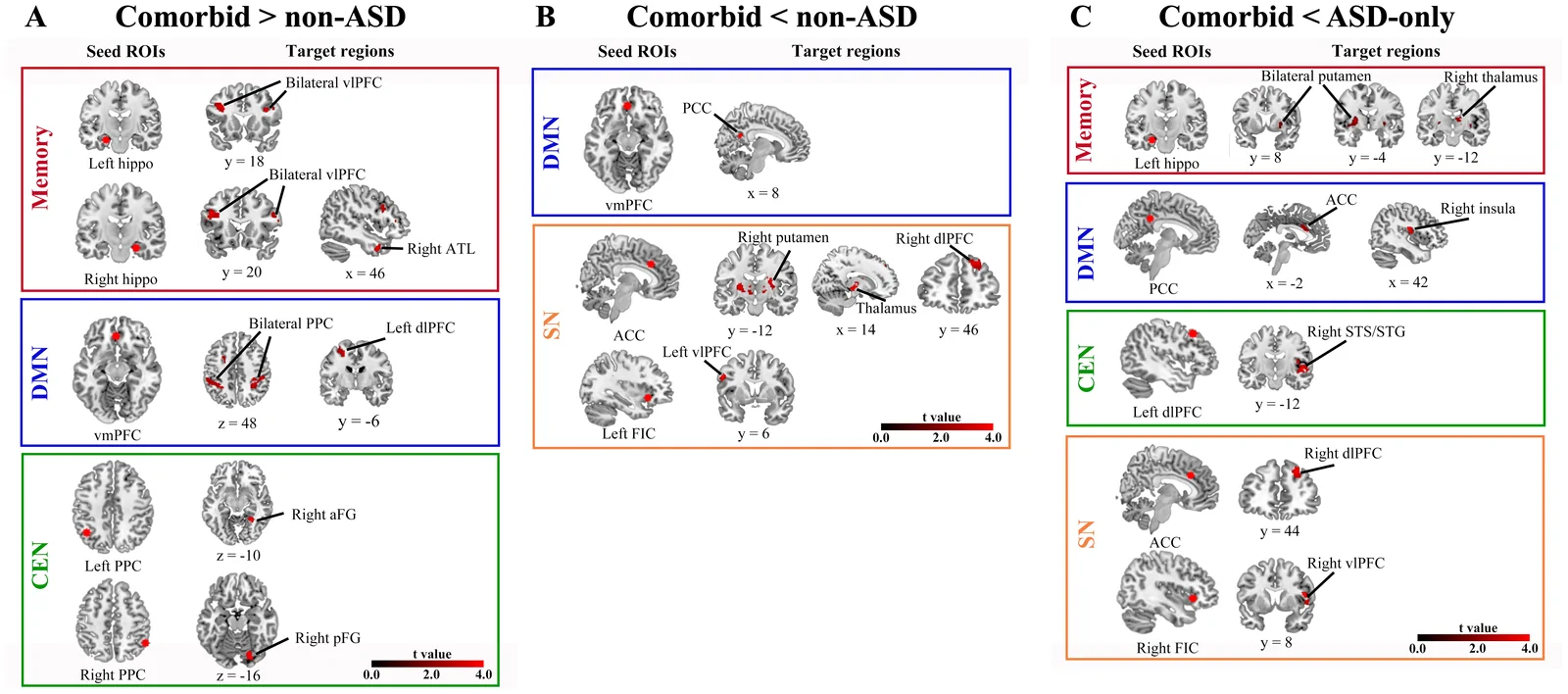
Brain regions showing hyper-connectivity with seed ROIs of Hippocampal-memory, DMN, and CEN network nodes in the Comorbid group compared to non-ASD **(A, B)** and ASD-only groups **(C)**. Hippo, hippocampus; vlPFC, ventrolateral prefrontal cortex; ATL, anterior temporal lobe; vmPFC, ventromedial prefrontal cortex; PPC, posterior parietal cortex; a/pFG, anterior/posterior fusiform gyrus; PCC/ACC, posterior/anterior cingulate cortex; dlPFC/vlPFC, dorsolateral/ventrolateral prefrontal cortex; FIC, fronto-insular cortex; STS/STG, superior temporal sulcus/gyrus.

Compared to non-ASD, autistic individuals with comorbid ADHD showed increased connectivity from the bilateral hippocampus ROIs to bilateral ventrolateral prefrontal cortex (vlPFC), from right hippocampus to right anterior temporal lobe (ATL), from the ventromedial PFC (vmPFC) to left dorsolateral PFC (dlPFC) and bilateral posterior parietal cortex (PPC), and from the left and right PPC to the right medial part of fusiform gyrus (FG; **Supplementary Table 1**; **Figure 2A**). At the same time, the Comorbid group showed decreased connectivity compared to the non-ASD group from the vmPFC to the posterior cingulate cortex (PCC), from the left fronto-insular cortex (FIC) to the vlPFC, and finally from the anterior cingulate cortex (ACC) to the right dlPFC, the thalamus, and the right putamen (**Supplementary Table 1**; **Figure 2B**). Amongst these differences, only the atypical connectivity between left hippocampus and vlPFC survived the stringent threshold at *p* <.001 for height threshold at the cluster level of *p* <.01.

Surprisingly, we did not observe any increased intrinsic functional connectivity in the Comorbid group compared to the ASD-only group, but did observe decreased connectivity from the left hippocampus ROI to putamen and thalamus, from the PCC to right insula and ACC, from the left dlPFC to superior temporal sulcus/gyrus, from ACC to right dlPFC, and from the right FIC to the vlPFC (see **Supplementary Table 2**; **Figure 2C**). Amongst all these differences, only the connectivity between the PCC and right insula survived the stringent threshold at *p* <.001 for height threshold at the cluster level of *p* <.01. Because the peaks of the right dlPFC identified for ACC in the Comorbid < ASD-only and non-ASD comparisons were very close, we only included R dlPFC (24,44,40) from the Comorbid < ASD-only for the following analysis.

#### Shared and distinct connectivity patterns in comorbid and ASD-only groups

**Figure 3:**
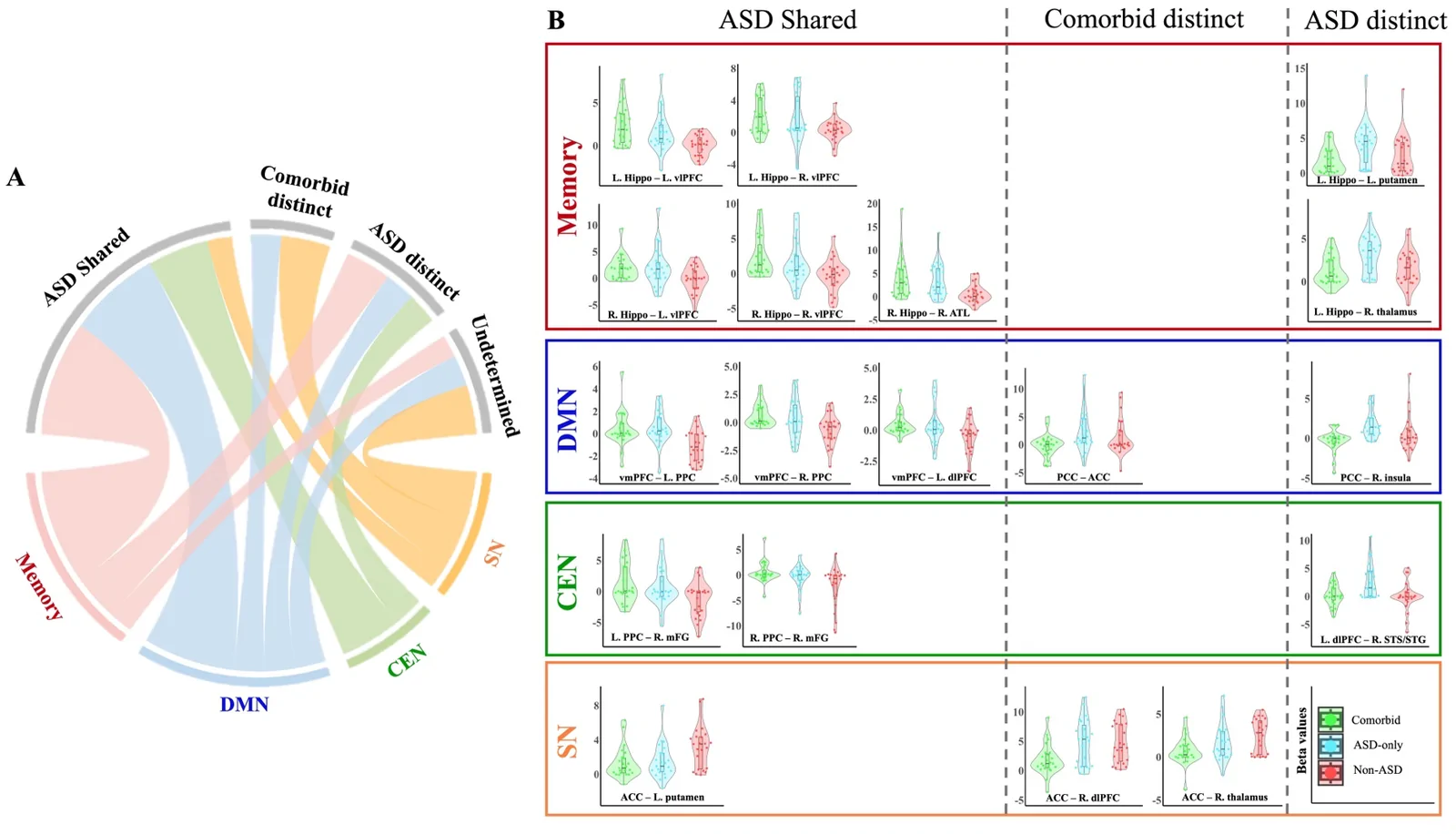
Classifications of observed atypical connectivity patterns in the Comorbid group compared to ASD-only and non-ASD groups. **(A)** Adjusted proportion (the total number of connectivity in each type divided by the total number of connectivity and adjusted by the number of seed ROIs in each network) of hippocampal-memory, DMN, CEN, and SN networks for the types of profiles. **(B)** Violin plots of beta values of classified connectivity profiles. Hippo, hippocampus, vlPFC, ventrolateral prefrontal cortex; ATL, anterior temporal lobe; vmPFC, ventromedial prefrontal cortex; PPC, posterior parietal cortex; PCC/ACC, posterior/anterior cingulate cortex; dlPFC, dorsolateral prefrontal cortex; mFG, medial fusiform gyrus; STS/STG, superior temporal sulcus/gyrus.

First of all, the significant results observed above showed no graded pattern in connectivity from any ROIs across the Comorbid, ASD-only, and non-ASD groups, failing to support an additive ASD-variant model. Not surprisingly, the majority of effects suggested an overlapping neurobiological basis in the Comorbid and ASD-only groups compared to their non-ASD peers. Across all networks we examined, the hippocampal-memory, DMN, and CEN showed similar patterns of ASD-relevant (both shared and distinct) effects, but the SN was more relevant to the Comorbid-distinct effect (**Figure 3A**). Specifically, the connectivity of hippocampal-memory, DMN, and CEN nodes showed increased connectivity, whereas the ACC node of the SN network was the only region that showed decreased connectivity in the Comorbid and ASD-only groups compared to non-ASD (for more details, see **Table 2**; **Figure 3B**). The Comorbid-distinct effect was observed only for the ACC node of the SN network with right dlPFC and thalamus, and with PCC node in DMN. Interestingly, we also observed ASD-distinct effect of all networks except SN, including left hippocampus with left putamen and right thalamus, PCC with right insula, and left dlPFC with right STS/STG. Within the hippocampal-memory network, the connectivity of both left and right hippocampus showed ASD-shared effects, but the ASD-distinct effect was observed only for the left hippocampus with subcortical regions. For DMN, only vmPFC showed ASD-shared effects, whereas the connectivity between PCC and right dorsal insula showed ASD-distinct effect. For nodes in CEN, bilateral PPC with right medial FG showed ASD-shared effect, whereas left dlPFC with right STS/STG showed ASD-distinct effect. For SN, ACC was the only seed that showed significant effects and was the only region with connectivity patterns showing Comorbid-distinct effects (**Figure 3B**). All the statistical results can be found in **Supplementary Table 3**.

**Table 2:** Resting-state functional connectivity with significant differences between the Comorbid and ASD-only or non-ASD groups.

Network	Source	Seed ROI	Target region	MNI coordinates (x, y, z)			ASD shared (comorbid = ASD-only ≠ non-ASD)	Comorbid distinct (comorbid ≠ ASD-only = non-ASD)	ASD distinct (ASD-only ≠ comorbid = non-ASD)	Undetermined
Memory	*Comorbid > non-ASD*	*L Hippo*	L vlPFC	-32	16	30	Y			
	*Comorbid > non-ASD*	*L Hippo*	R vlPFC	38	18	26	P			
	*Comorbid < ASD-only*	*L Hippo*	L Putamen	-26	-4	6			P	
	*Comorbid < ASD-only*	*L Hippo*	R Putamen	28	8	2				Y
	*Comorbid < ASD-only*	*L Hippo*	R Thalamus	14	-12	8			Y	
	*Comorbid > non-ASD*	*R Hippo*	R vlPFC	50	16	26	P			
	*Comorbid > non-ASD*	*R Hippo*	L vlPFC	-44	20	30	Y			
	*Comorbid > non-ASD*	*R Hippo*	R ATL	46	6	-34	Y			
DMN	*Comorbid < ASD-only*	*PCC*	R Insula	42	-12	18			P	
	*Comorbid < ASD-only*	*PCC*	ACC	-2	16	26		Y		Y
	*Comorbid > non-ASD*	*vmPFC*	R PPC	46	-32	48	P			
	*Comorbid > non-ASD*	*vmPFC*	L PPC	-26	-50	46	Y			
	*Comorbid > non-ASD*	*vmPFC*	L dlPFC	-24	-6	54	Y			
	*Comorbid < non-ASD*	*vmPFC*	PCC	8	-54	22				Y
CEN	*Comorbid < ASD-only*	*L dlPFC*	R STS/STG	50	-12	0			Y	
	*Comorbid > non-ASD*	*L PPC*	R medial aFG	28	-50	-10	P			
	*Comorbid > non-ASD*	*R PPC*	R medial pFG	20	-74	-16	P			
SN	*Comorbid < ASD-only*	*ACC*	R dlPFC	24	44	40		Y		
	*Comorbid < non-ASD*	*ACC*	R dlPFC	24	46	40				
	*Comorbid < non-ASD*	*ACC*	L Putamen	-26	-12	8	Y			
	*Comorbid < non-ASD*	*ACC*	R Thalamus	14	-18	-4		Y		
	*Comorbid < non-ASD*	*L FIC*	L vlPFC	-54	6	30				Y
	*Comorbid < ASD-only*	*R FIC*	R vlPFC	48	8	16				Y

Hippo, hippocampus; vlPFC, ventrolateral prefrontal cortex; ATL, anterior temporal lobe; aFG/pFG, anterior/posterior fusiform gyrus; PPC, posterior parietal cortex; vmPFC, ventromedial prefrontal cortex; dlPFC, dorsolateral prefrontal cortex; STS/STG, superior temporal sulcus/gyrus; FIC, fronto-insular cortex; ACC, anterior cingulate cortex; PCC, posterior cingulate cortex. Y, classified effects with significant results for *post-hoc* tests (*ps* <.05); P, classified effects with trending effects for *post-hoc* tests (*ps* <.10). Because the peaks of the right dlPFC identified for ACC in the Comorbid < ASD-only and non-ASD were very close, we only included R dlPFC (24,44,40) from the Comorbid < ASD-only for the following analysis.

#### Atypical connectivity patterns in comorbid and ASD-only groups predicting ASD symptoms

**Figure 4:**
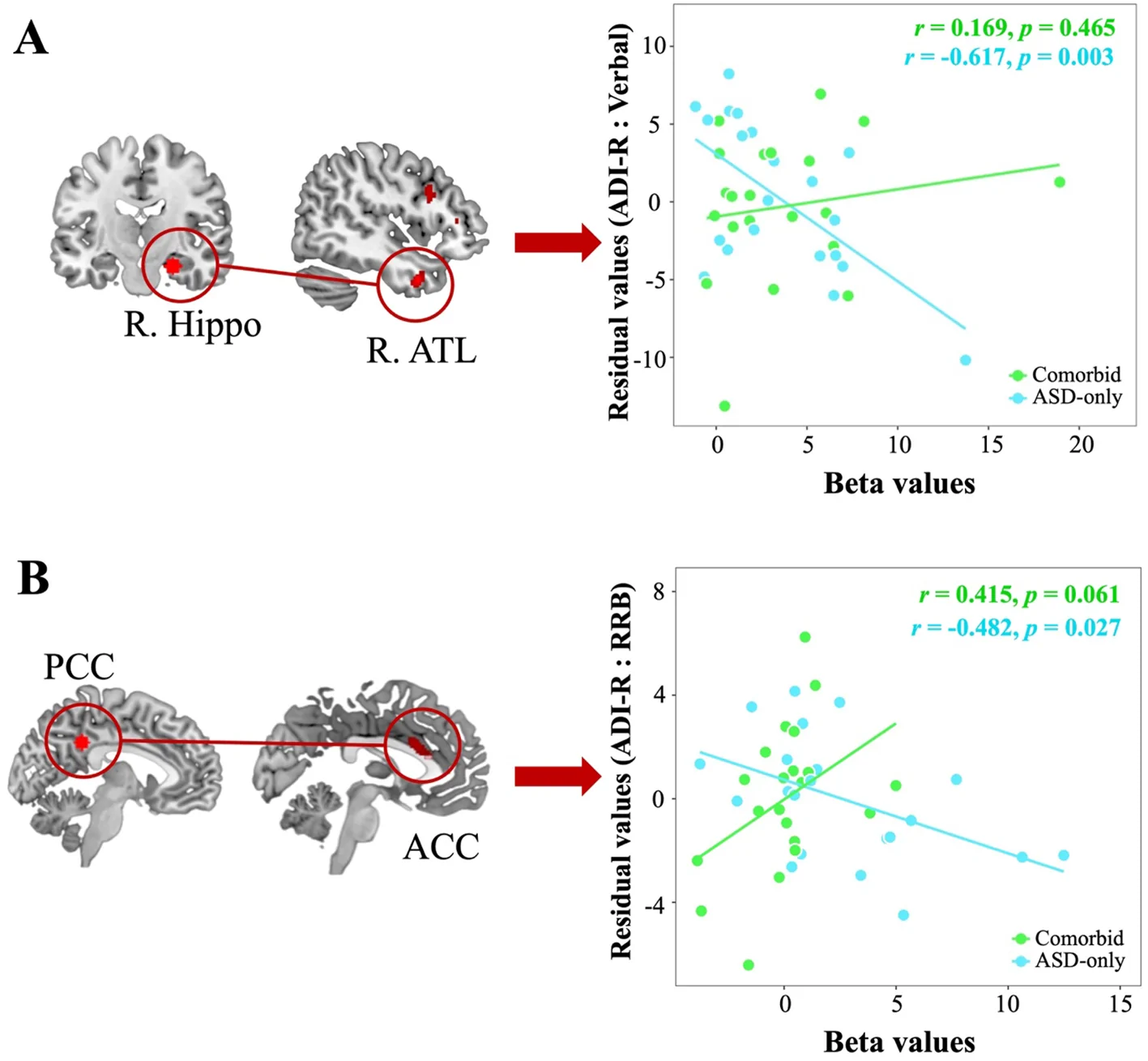
The connectivity in the ASD-only group, but not in the Comorbid group, was associated with ASD symptom severity. **(A)** Beta values of the connectivity between the right hippocampus and right anterior temporal lobe (ATL) predicted the verbal communication subscales scores when the effects of age, gender, FSIQ, and movement in the scan were controlled for. **(B)** Beta values of the connectivity between PCC and ACC predicted the RRB subscale scores.

After controlling for the effects of age, gender, FSIQ, and movement parameters in the scan, we observed a significant main effect of the connectivity between the right hippocampus and ATL (ASD-shared effect) in predicting the Verbal communication subscale score, B = -0.90, *t* = -3.07, *p* = 0.004 (FDR-adjusted *p* = 0.046), and a significant interaction between the Group (Comorbid vs. ASD-only) and the connectivity of PCC and ACC (Comorbid-distinct effect) on predicting the Restricted, repetitive behavior subscale scores in ADI-R, B = 0.91, *t* = 2.79, *p* = 0.009 (FDR-adjusted *p* = 0.06; See **Figure 4**; **Table 3**). For the connectivity between the right Hippocampus and ATL, the relationship was also modulated by the Group, B = 1.03, t = 2.82, *p* = 0.008 (FDR-adjusted *p* = 0.087), showing a significant negative relationship between connectivity strength and symptom severity in the ASD-only group, *r*(19) = -0.617, *p* = 0.003, but not in the Comorbid group, *r*(19) = 0.169, *p* = 0.465. This result remained significant when the two participants with extremely high beta values were excluded (see **Supplementary Figure 1**). A permutation test showed that these connectivity-behavior associations were reliable as well. For the connectivity between PCC and ACC, there was a negative relationship between connectivity strength and symptom severity in the ASD-only group, *r*(19) = -0.482, *p* = 0.027, but a trend for a positive relationship in the Comorbid group, *r*(19) = 0.415, *p* = 0.061. To test the reliability of these effects, we ran additional permutation tests and found that these significant interactions were reliable (both *p*s *<*0.005), and the negative correlations between connectivity and ASD symptoms in the ASD-only group were also unlikely due to chance (both *p*s *<*0.018). More details can be found in the **Supplementary Methods and Results** (**Supplementary Figures 2, 3**; **Supplementary Table 4**).

**Table 3:** Multiple regression results for the connectivity predicting ADI-R subscale scores in Comorbid and ASD-only groups.

Connectivity	ADI-R subscale	Main effect (connectivity)	Interaction (connectivity*group)
R.Hippo - R. ATL	Verbal	B = -0.90, *t* = -3.07, p = 0.004	B = 1.03, *t* = 2.82, *p* = 0.008
PCC - ACC	RRB	B = -0.30, t = -1.96, p = 0.058	B = 0.91, t = 2.79, p = 0.009

Hippo, hippocampus; ATL, anterior temporal lobe; vlPFC, ventrolateral prefrontal cortex; PCC, posterior cingulate cortex. All the results were controlled for the effect of age, gender, FSIQ, and the movement parameters in the scanner.

## Discussion

With Comorbid, ASD-only, and non-ASD groups that were well-matched on gender, age, FSIQ, and movement in the fMRI scan, we revealed both shared and distinct resting-state functional connectivity (RSFC) patterns of the hippocampal-memory, DMN, CEN, and SN nodes. Specifically, hippocampal-memory, DMN, and CEN nodes mostly showed shared RSFC features in the Comorbid and ASD-only groups compared to the non-ASD group, as well as distinct patterns of RSFC only found in the ASD-only group. By contrast, the RSFC of ACC in SN was associated with Comorbid-distinct patterns. Moreover, in the ASD-only group, RSFC between the right hippocampus and ATL was negatively related to verbal communication ability, and the RSFC between PCC and ACC was also negatively correlated with repetitive, restricted behaviors. These relationships were not found in the Comorbid group. Therefore, the absence of an additive effect and the presence of the ASD-distinct effect (**Figure 1B**) in addition to a unique brain-behavior relationship in the ASD-only group, our findings support a non-additive ASD-variant model of the Comorbid ASD+ADHD condition.

Our findings were consistent with the triple-network theory (10, 11), supporting the pivotal role of the DMN, CEN, and SN in neurodevelopmental disorders, such as ASD. Specifically, the current study demonstrated that autistic individuals with or without comorbid ADHD shared increased RSFC of triple-network nodes compared to their non-autistic peers. The increased RSFC of vmPFC with left dlPFC and bilateral PPC in autistic individuals is possibly associated with their poor cognitive flexibility (66). Studies have revealed that the connectivity between vmPFC and nodes in left fronto-parietal regions was correlated with cognitive flexibility (67), value-based decision-making (68, 69), and transfer ability in learning (70) in non-autistic individuals. Thus, our results further highlight the importance of vmPFC-CEN connectivity for cognitive skills in affected individuals. We also observed hyper-connectivity between the bilateral PCC and the right medial FG in both Comorbid and ASD-only groups, suggesting their potential challenges in object recognition (71) due to integration of information from ventral and dorsal visual streams (72) or attention control for perceptual processing (73, 74). The ACC node of SN was the only node showing hypo-connectivity with PCC, putamen, thalamus, and right dlPFC in autistic individuals compared to non-autistic individuals, and all the Comorbid-distinct effects (i.e., Comorbid < ASD-only = non-ASD) were observed for its connectivity. The ACC is commonly associated with inhibitory control and response monitoring (75–77), and hypo-connectivity of the ACC in ASD has been reported (78, 79). Therefore, the underconnectivity in autistic individuals, especially those with comorbid ADHD, may be linked to their difficulties in attention and inhibition control (80).

Besides the triple networks, we examined the RSFC of the hippocampal nodes, given the recent theories and findings of hippocampal dysfunctions in ASD (81, 82) and their challenges in episodic memory tasks (55, 56, 83). Interestingly, both Comorbid and ASD-only groups showed increased RSFC of bilateral hippocampal nodes compared to the non-ASD group. The aberrant connectivity was mostly with the vlPFC, which is commonly involved in verbal memory (84, 85), and the ATL, which is considered a cross-modal hub for semantic memory (86–88). Thus, the aberrant connectivity of the bilateral hippocampus further suggests hippocampal dysfunctions and memory challenges in ASD. They are as critical as the dysfunctions of the triple networks and socio-communicative difficulties. More importantly, we observed that the RSFC between the left hippocampal node and the putamen and thalamus was particularly elevated in the ASD-only group compared to the Comorbid and non-ASD groups. This implies some dysfunctions in motivated learning (89, 90) and sensory integration (91, 92) underlying the memory challenges in autistic individuals. It is noteworthy that the hippocampus has been suggested as part of the DMN (58, 93), and therefore, our findings of hippocampal RSFC could be interpreted as supporting evidence on the triple-network model for ASD (11).

Our exploratory brain-behavior analysis further revealed two critical links for their relevance to ASD symptoms. The first was the right hippocampal-ATL connectivity with verbal communication abilities. As discussed above, the hippocampal-ATL connectivity could be related to the interaction between episodic memory and semantic memory for the key roles of the hippocampus and ATL (88, 94–97). Interestingly, there was only a negative relationship observed in the ASD-only group, suggesting that the increased connectivity between the right hippocampus and ATL was associated with milder symptoms in verbal communications. Thus, the strong intrinsic connectivity between these two regions could function as a compensatory mechanism or a protective factor for their verbal abilities. Surprisingly, such an effect was not observed in the Comorbid group. A similar pattern was observed for the PCC-ACC connectivity and repetitive, restricted behaviors (RRB) in the ASD-only group, so that the increased RSFC between PCC and ACC may protect affected individuals from severe symptoms of RRB. Interestingly, overall, the Comorbid group had lower connectivity between these two regions compared to the ASD-only group, and the opposite relationship was observed for autistic individuals with comorbid ADHD. Given the important role of ACC in attention and response control (76, 77), the increased PCC-ACC connectivity within the low range was detrimental for their RRB symptoms with an additional ADHD condition. These brain-behavior relations further showed that the ASD-only and Comorbid groups may have distinct underlying mechanisms for their behavioral symptoms.

By examining the RSFC of the DMN, CEN, SN, and hippocampal nodes, we observed shared connectivity patterns in the Comorbid and ASD-only groups, but also distinct patterns for each group compared to non-ASD (**Figure 1B**). Since there was no additive effect was observed, our findings support the non-additive ASD-variant model (**Figure 1A**) for the comorbid condition in ASD. Our results are consistent with a few behavioral studies (5, 52), as well as the majority of the neuroimaging studies (40, 48, 53). However, there was indeed a large body of literature showing more severe cognitive impairments or symptoms in autistic individuals with a comorbid ADHD (4, 49–51). Two possibilities could explain the discrepancy. First, our Comorbid and ASD-only groups were well-matched on gender, age, and FSIQ, and they did not differ in their ASD symptoms as a result of the matching. Therefore, it is possible that we failed to observe any additive effects on RSFC because their cognitive and symptom profiles were very similar to each other. Second, the additive effects may be manifested only when autistic individuals are engaged in cognitive processes. If so, the additive effects will be observed during task-based fMRI measures and behavioral assessments with active tasks for their cognitive abilities or symptom outcomes. Future studies should examine the differences between resting-state and task-based connectivity to adjudicate between different models.

Although the current study showed evidence to support the non-additive ASD-variant model of ASD with comorbid ADHD, a few limitations should be taken into consideration. First, our study only examined the RSFC and its relationship to ASD symptoms, so future studies should use other behavioral or neurobiological measures to further adjudicate between these models. Secondly, our study did not include an ADHD-only group, so it is unclear how the comorbid group could be different from their single-diagnosis counterparts (37, 40, 44). Future studies should further examine the comorbid condition compared to both ASD-only and ADHD-only conditions. Also, our sample size was relatively small (n = 23), so some potential group differences and connectivity-behavior associations went undetected. Particularly, if the additive effect was not observed because of low power, our suggestion of the non-additive model should be reconsidered. Last, future studies should use other independent datasets such as ABIDE-II to replicate our findings and use different approaches and measures to examine the differences between autistic individuals with and without comorbid ADHD (46, 98).

In conclusion, our study revealed both shared and distinct patterns of resting-state functional connectivity of the hippocampal memory, DMN, CEN, and SN nodes in autistic individuals with or without comorbid ADHD. Our results suggest that the comorbid condition should be considered as a variant of ASD instead of a subtype. Furthermore, the hippocampal network seems to be critical for our understanding of ASD. It should be examined more in future research for its role in memory functions and also in the DMN. If the comorbid ASD and ADHD condition is indeed neurobiologically different from ASD, future clinical and educational practices may need to have specialized programs to support affected individuals with or without comorbid ADHD, and pay more attention to their memory functions in addition to their socio-communicative challenges.

## Data Availability

The original contributions presented in the study are included in the article/**Supplementary Material**. Further inquiries can be directed to the corresponding author.
