# Supplementary material for: Overlapping and distinct intrinsic connectivity of hippocampal and triple networks in autistic individuals with and without a comorbid condition of ADHD
Source: Front Psychiatry. 2026 Sep 3;17:1894578. doi: 10.3389/fpsyt.2026.1894578 (PMC13581912; doi:10.3389/fpsyt.2026.1894578)
Supplement: Supplementary file 1 [file Table1.docx]

**Supplementary Materials**

*Overlapping and distinct intrinsic connectivity in autistic individuals with and without ADHD*

***Table of contents***

*Supplementary Method*

*Supplementary Results*

*Supplementary Figures S1-S3*

*Supplementary Tables S1-S4*

**Supplementary Method**

*Permutation tests to assess the reliability of the connectivity-behavior relationship*

Since the sample size was small in the ASD-only and Comorbid groups (n = 21 in each group), we ran additional permutation tests to assess the reliability of the association between the connectivity and ASD symptoms. In each permutation, we shuffled the values of every variable used in the analysis. Specifically, in the regression analysis to assess the main effect of connectivity and the interaction between connectivity and group, the gender, age, FSIQ, mean framewise displacement (FD), beta values of connectivity, and ADI-R subscale scores were reshuffled across all participants. Then, the t-values of the main effect of connectivity and the interaction between connectivity and group were calculated, when the effects of gender, age, FSIQ, and mean FD were controlled for. The t-values for the correlation between connectivity beta values and residual ADI-R scores in ASD-only and Comorbid groups were calculated as well. After 1,000 permutations, we calculated the percentiles of the observed t-values in our data in the permutated distributions of main effect, interaction, and correlations in ASD-only and correlation in Comorbid groups, respectively. These percentiles were used as the *p*-values to indicate whether the effects were reliable or occurred due to chance.

**Supplementary Results**

*Reliable results of the connectivity-behavior relationship*

Our permutation tests showed that the main effects of connectivity and interactions between connectivity and group were reliable for both the R.hippo-ATL and PCC-ACC connectivity (**Figures S2-S3** and **Table S4**). The t-value observed for R. hippo-ATL connectivity and verbal communication subscale score was -3.071, significantly lower than the permutated mean t-value, -0.026, *p* = 0.001. The same was observed for the interaction between connectivity and group, *p* = 0.003. We also found that the negative correlation in the ASD-only group was reliable, *p* = 0.001, and the correlation in the Comorbid group was also reliably insignificant, *p* = 0.237. Similar results were found for the PCC-ACC connectivity and RRB subscale score for main effect and interaction, both *p*s < 0.05, and the different correlations in ASD-only and Comorbid groups were also found to be significant, both *p*s < 0.05.

**Figure S1**. Association between right hippocampus-ATL connectivity and subscale scores of verbal communication in ADI-R when two individuals with extremely large beta values were removed.

**Figure S2**. Histograms of the t-value distributions in the permutation test to assess the reliability of the main effect of Connectivity and the interaction between Group and Connectivity (right hippocampus-ATL) on ADI-R verbal communication subscale score. (**A**) T-values for the main effect of Group, (B) t-values for the interaction of Group and Connectivity, and (**C**) t-values for the correlation between connectivity and verbal communication subscale score in ASD-only and Comorbid groups. The dashed lines show the mean values of 1,000 permutations for the t-values, and the solid lines mark the t-values observed in the current study.

**Figure S3**. Histograms of the t-value distributions in the permutation test to assess the reliability of the main effect of Connectivity and the interaction between Group and Connectivity (PCC-ACC) on ADI-R RRB subscale score. (**A**) T-values for the main effect of Group, (B) t-values for the interaction of Group and Connectivity, and (**C**) t-values for the correlation between connectivity and verbal communication subscale score in ASD-only and Comorbid groups. The dashed lines show the mean values of 1,000 permutations for the t-values, and the solid lines mark the t-values observed in the current study.

| **Table S1**. Resting-state functional connectivity differences of different ROIs between the Comorbid group and TD. | | | | | | | |
| --- | --- | --- | --- | --- | --- | --- | --- |
| Cluster | Brain regions | MNI Coordinates  (x, y, z) | | | cluster size  (# of voxels) | Z value | Survived threshold  (height, cluster) |
| ***Comorbid > TD*** | | | | | | | |
| *Left Hippocampus* |  |  |  |  |  |  |  |
| 1 | L vlPFC | -32 | 16 | 30 | 59 | 3.81 | **< 0.001, <0.01** |
| 2 | R vlPFC | 38 | 18 | 26 | 191 | 3.53 | < 0.01, <0.01 |
| *Right Hippocampus* |  |  |  |  |  |  |  |
| 1 | R vlPFC | 50 | 16 | 26 | 300 | 3.56 | < 0.01, <0.01 |
| 2 | L vlPFC | -44 | 20 | 30 | 212 | 3.48 | < 0.01, <0.01 |
| 3 | R ATL | 46 | 6 | -34 | 131 | 3.49 | < 0.01, <0.01 |
| *Left PPC* |  |  |  |  |  |  |  |
| 1 | R medial FG | 28 | -50 | -10 | 129 | 3.13 | < 0.01, <0.01 |
| *Right PPC* |  |  |  |  |  |  |  |
| 1 | R medial FG | 20 | -74 | -16 | 158 | 3.01 | < 0.01, <0.01 |
| *vmPFC* |  |  |  |  |  |  |  |
| 1 | R PPC | 46 | -32 | 48 | 509 | 3.87 | < 0.01, <0.01 |
| 2 | L PPC | -26 | -50 | 46 | 265 | 3.33 | < 0.01, <0.01 |
| 3 | L dlPFC | -24 | -6 | 54 | 208 | 3.21 | < 0.01, <0.01 |
| ***TD > Comorbid*** | | | | | | | |
| *ACC* |  |  |  |  |  |  |  |
| 1 | R dlPFC | 24 | 46 | 40 | 205 | 3.29 | < 0.01, <0.01 |
| 2 | R putamen | -26 | -12 | 8 | 307 | 3.2 | < 0.01, <0.01 |
| 3 | R thalamus | 14 | -18 | -4 | 500 | 3.47 | < 0.01, <0.01 |
| *Left FIC* |  |  |  |  |  |  |  |
| 1 | L vlPFC | -54 | 6 | 30 | 161 | 3.3 | < 0.01, <0.01 |
| *vmPFC* |  |  |  |  |  |  |  |
| 1 | PCC | 8 | -54 | 22 | 137 | 2.87 | < 0.01, <0.01 |

*Notes*: vlPFC = ventrolateral prefrontal cortex; ATL = anterior temporal lobe; FG = fusiform gyrus; PPC = posterior parietal cortex; vmPFC = ventromedial prefrontal cortex; dlPFC = dorsolateral prefrontal cortex; FIC = fronto-insular cortex; ACC = anterior cingulate cortex; PCC = posterior cingulate cortex.

| **Table S2**. Resting-state functional connectivity differences of different ROIs between Comorbid group and ASD-only group. | | | | | | | |
| --- | --- | --- | --- | --- | --- | --- | --- |
| Cluster | Brain regions | MNI Coordinates  (x, y, z) | | | cluster size  (# of voxels) | Z value | Survived threshold  (height, cluster) |
| ***Comorbid > ASD*** | | | | | | | |
| *No significant results* |  |  |  |  |  |  |  |
| ***ASD > Comorbid*** |  |  |  |  |  |  |  |
| *Left Hippocampus* |  |  |  |  |  |  |  |
| 1 | L Putamen | -26 | -4 | 6 | 229 | 3.65 | < 0.01, <0.01 |
| 2 | R Putamen | 28 | 8 | 2 | 138 | 2.38 | < 0.01, <0.01 |
| 3 | R Thalamus | 14 | -12 | 8 | 135 | 3.42 | < 0.01, <0.01 |
| *ACC* |  |  |  |  |  |  |  |
| 1 | R dlPFC | 24 | 44 | 40 | 176 | 3.27 | < 0.01, <0.01 |
| *Left dlPFC* |  |  |  |  |  |  |  |
| 1 | R STS/STG | 50 | -12 | 0 | 413 | 3.36 | < 0.01, <0.01 |
| *PCC* |  |  |  |  |  |  |  |
| 1 | R Insula | 42 | -12 | 18 | 103 | 4.32 | **< 0.001, <0.01** |
| 2 | ACC | -2 | 16 | 26 | 163 | 3.09 | < 0.01, <0.01 |
| *Right FIC* |  |  |  |  |  |  |  |
| 1 | R vlPFC | 48 | 8 | 16 | 175 | 3.2 | < 0.01, <0.01 |

*Notes*: vlPFC = ventrolateral prefrontal cortex; dlPFC = dorsolateral prefrontal cortex; STS/STG = superior temporal sulcus/gyrus; FIC = fronto-insular cortex; ACC = anterior cingulate cortex; PCC = posterior cingulate cortex.

| **Table S3.** ANCOVA and post-hoc results to compare the beta values of the connectivity across three groups to classify the profiles. | | | | | | | | | |
| --- | --- | --- | --- | --- | --- | --- | --- | --- | --- |
| **Seed ROI** | **Target region** | **Profiles** | **Main effect (Group)** | **Comorbid**  **Mean (SD)** | **ASD-only**  **Mean (SD)** | **non-ASD**  **Mean (SD)** | **Comorbid**  **vs.**  **ASD-only** | **Comorbid**  **vs.**  **non-ASD** | **ASD-Only vs.**  **non-ASD** |
| *Left Hippocampus* | L vlPFC | ASD Shared | *F*(2,62) = 9.728** | 2.39 (2.34) | 1.60 (2.34) | 0.13 (1.16) | *t*(44) = 1.14 | ***t*(44) = 4.15***** | ***t*(44) = 2.71**** |
| *Left Hippocampus* | R vlPFC | ASD Shared | *F*(2,62) = 4.156* | 2.04 (2.27) | 1.65 (3.19) | 0.28 (1.37) | *t*(44) = 0.48 | ***t*(44) = 3.18**** | ***t*(44) = 1.89#** |
| *Left Hippocampus* | L Putamen | ASD Distinct | *F*(2,62) = 5.130* | 1.82 (1.87) | 4.17 (3.12) | 2.59 (2.87) | ***t*(44) = -3.1**** | *t*(44) = -1.08 | ***t*(44) = 1.79#** |
| *Left Hippocampus* | R Putamen | Undetermined | *F*(2,62) = 3.589* | 1.92 (2.16) | 3.90 (3.17) | 2.91 (2.77) | ***t*(44) = -2.47*** | *t*(44) = -1.35 | *t*(44) = 1.13 |
| *Left Hippocampus* | R Thalamus | ASD Distinct | *F*(2,62) = 6.552** | 1.32 (1.67) | 3.17 (2.24) | 1.59 (2.07) | ***t*(44) = -3.19**** | *t*(44) = -0.49 | ***t*(44) = 2.49*** |
| *Right Hippocampus* | L vlPFC | ASD Shared | *F*(2,62) = 5.501* | 1.90 (2.25) | 2.05 (3.63) | -0.21 (2.44) | *t*(44) = -0.18 | ***t*(44) = 3.04**** | ***t*(44) = 2.48*** |
| *Right Hippocampus* | R vlPFC | ASD Shared | *F*(2,62) = 5.619* | 2.32 (2.97) | 1.47 (3.14) | -0.22 (2.34) | *t*(44) = 0.94 | ***t*(44) = 3.22**** | ***t*(44) = 2.07*** |
| *Right Hippocampus* | R ATL | ASD Shared | *F*(2,62) = 6.585** | 4.01 (4.49) | 3.38 (3.59) | 0.73 (2.04) | *t*(44) = 0.53 | ***t*(44) = 3.19**** | ***t*(44) = 3.07**** |
| *PCC* | R Insula | ASD Distinct | *F*(2,62) = 7.609** | -0.32 (1.40) | 1.72 (1.7) | 0.56 (2.29) | ***t*(44) = -4.44***** | *t*(44) = -1.58 | ***t*(44) = 1.94#** |
| *PCC* | ACC | Comorbid Distinct | *F*(2,62) = 4.124* | -0.09 (2.03) | 2.52 (3.89) | 1.41 (3.26) | ***t*(44) = -2.84**** | ***t*(44) = -1.87#** | *t*(44) = 1.05 |
| *vmPFC* | L PPC | ASD Shared | *F*(2,62) = 8.332** | 0.40 (1.57) | 0.51 (1.49) | -1.11 (1.40) | *t*(44) = -0.24 | ***t*(44) = 3.45**** | ***t*(44) = 3.80***** |
| *vmPFC* | R PPC | ASD Shared | *F*(2,62) = 4.838* | 0.60 (1.01) | 0.33 (1.79) | -0.63 (1.36) | *t*(44) = 0.63 | ***t*(44) = 3.47**** | ***t*(44) = 2.04*** |
| *vmPFC* | L dlPFC | ASD Shared | *F*(2,62) = 4.422* | 0.48 (0.90) | 0.44 (1.62) | -0.52 (1.33) | *t*(44) = 0.11 | ***t*(44) = 3.00**** | ***t*(44) = 2.20*** |
| *vmPFC* | PCC | Undetermined | *F*(2,62) = 4.741* | 1.87 (2.74) | 3.17 (3.31) | 4.5 (3.81) | *t*(44) = -1.45 | ***t*(44) = -2.68*** | *t*(44) = -1.26 |
| *Left dlPFC* | R STS/STG | ASD Distinct | *F*(2,62) = 7.634** | 0.21 (1.91) | 2.67 (3.07) | -0.004 (2.61) | ***t*(44) = -3.26**** | *t*(44) = 0.32 | ***t*(44) = 3.18**** |
| *Left PPC* | R medial aFG | ASD Shared | *F*(2,62) = 4.980* | 1.29 (3.18) | 0.74 (3.14) | -1.26 (2.77) | *t*(44) = 0.59 | ***t*(44) = 2.90**** | ***t*(44) = 2.29*** |
| *Right PPC* | R medial pFG | ASD Shared | *F*(2,62) = 5.585* | 0.51 (2.10) | -0.33 (2.41) | -2.36 (4.06) | *t*(44) = 1.26 | ***t*(44) = 3.01**** | ***t*(44) = 2.06*** |
| *ACC* | R dlPFC | Comorbid Distinct | *F*(2,62) = 6.185** | 1.86 (2.63) | 4.83 (3.93) | 4.71 (3.36) | ***t*(44) = -3.01**** | ***t*(44) = -3.21**** | *t*(44) = 0.11 |
| *ACC* | R dlPFC | Comorbid Distinct | *F*(2,62) = 6.322** | 1.74 (2.70) | 4.77 (3.97) | 4.65 (3.22) | ***t*(44) = -3.02**** | ***t*(44) = -3.31**** | *t*(44) = 0.11 |
| *ACC* | L Putamen | ASD Shared | *F*(2,62) = 6.145** | 1.34 (1.80) | 1.50 (2.03) | 3.16 (2.46) | *t*(44) = -0.29 | ***t*(44) = -2.88**** | ***t*(44) = -2.50*** |
| *ACC* | R Thalamus | Comorbid Distinct | *F*(2,62) = 5.898* | 0.71 (1.61) | 1.76 (2.15) | 2.48 (1.98) | ***t*(44) = -1.88#** | ***t*(44) = -3.33**** | *t*(44) = -1.18 |
| *Left FIC* | L vlPFC | Undetermined | *F*(2,62) = 2.749# | 1.57 (1.97) | 2.49 (3.48) | 3.37 (2.48) | *t*(44) = -1.11 | ***t*(44) = -2.74**** | *t*(44) = -0.99 |
| *Right FIC* | R vlPFC | Undetermined | *F*(2,62) = 4.249* | 2.12 (2.03) | 4.12 (2.79) | 2.92 (2.33) | ***t*(44) = -2.79**** | *t*(44) = -1.25 | *t*(44) = 1.59 |

*Notes:* For the main effect of Group, the original degree of freedom for the ANCOVA test was reported, but the p-values were adjusted by the FDR method, #<0.10, *<0.05, **<0.01. The post hoc t-tests between groups were used to classify the type of effects (ASD-shared, ASD-distinct, Comorbid-distinct, and undetermined).

| **Table S4**. Permutation results for the association between connectivity and ASD symptoms. | | | | | |
| --- | --- | --- | --- | --- | --- |
|  |  | Main Effect (Connectivity) | Interaction (Group*Connectivity) | Correlation (Comorbid) | Correlation (ASD) |
| R. Hippo | Permuted mean *t* | -0.026 | -0.018 | -0.053 | -0.023 |
|  | Observed *t* | -3.071 | 2.823 | 0.746 | -3.417 |
|  | *p* | **0.001** | **0.003** | 0.237 | **0.001** |
| PCC | permuted mean *t* | -0.042 | 0.040 | 0.024 | -0.036 |
|  | Observed *t* | -1.964 | 2.793 | 1.990 | -2.397 |
|  | *p* | **0.033** | **0.005** | **0.032** | **0.018** |
